# Supplementary figures and images for: Optimised biomolecular extraction for metagenomic analysis of microbial biofilms from high-mountain streams
Source: PeerJ. 2020 Oct 27;8:e9973. doi: 10.7717/peerj.9973 (PMC7597623; doi:10.7717/peerj.9973)

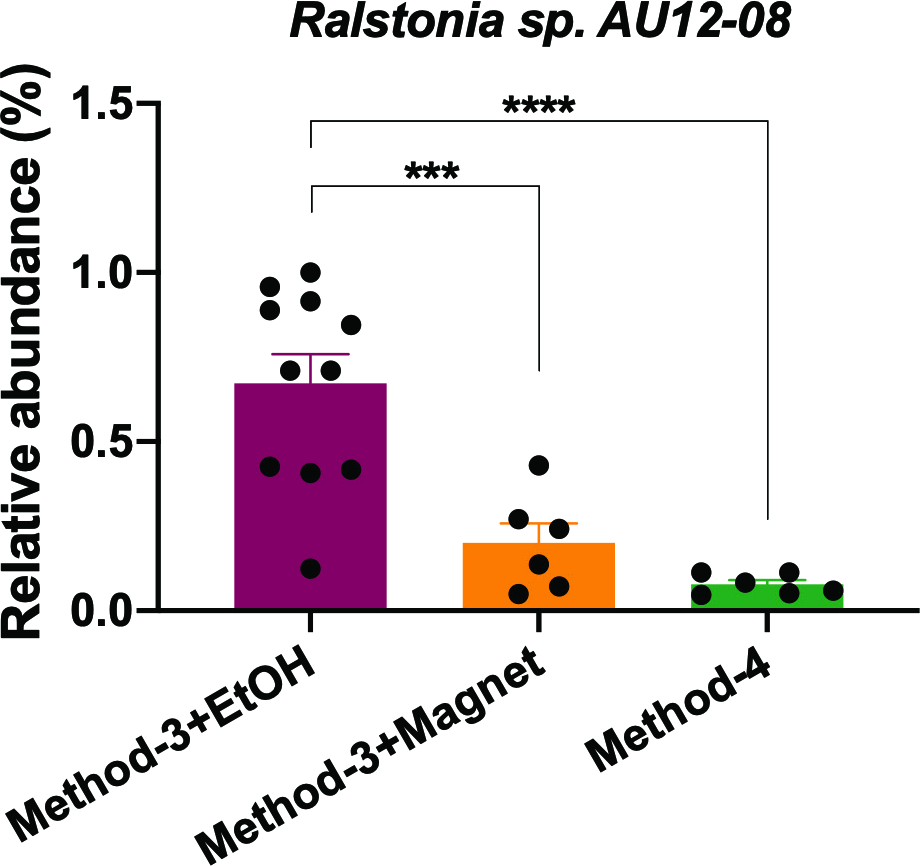

Supplement: Supplemental Information 2 — The abundance of the Ralstonia genome recovered from the samples when processed with method-3 (EtOH and magnetic bead clean-up) and method-4. Significance was tested using One-Way ANOVA with Student-Neuman Keul’s post-hoc analyses. ∗∗∗p < 0.001, ∗∗∗∗p < 0.0001. [file peerj-08-9973-s002.png]
